# Zero-Shot Action Recognition with Three-Stream Graph Convolutional Networks †

**DOI:** 10.3390/s21113793

**Published:** 2021-05-30

**Authors:** Nan Wu, Kazuhiko Kawamoto

**Affiliations:** 1 Department of Applied and Cognitive Informatics, Graduate School of Science and Engineering, Chiba University, Chiba 263-8522, Japan; gonan@chiba-u.jp; 2 Graduate School of Engineering, Chiba University, Chiba 263-8522, Japan

**Keywords:** deep learning, zero-shot learning (ZSL), zero-shot action recognition (ZSAR), DeViSE

## Abstract

Large datasets are often used to improve the accuracy of action recognition. However, very large datasets are problematic as, for example, the annotation of large datasets is labor-intensive. This has encouraged research in zero-shot action recognition (ZSAR). Presently, most ZSAR methods recognize actions according to each video frame. These methods are affected by light, camera angle, and background, and most methods are unable to process time series data. The accuracy of the model is reduced owing to these reasons. In this paper, in order to solve these problems, we propose a three-stream graph convolutional network that processes both types of data. Our model has two parts. One part can process RGB data, which contains extensive useful information. The other part can process skeleton data, which is not affected by light and background. By combining these two outputs with a weighted sum, our model predicts the final results for ZSAR. Experiments conducted on three datasets demonstrate that our model has greater accuracy than a baseline model. Moreover, we also prove that our model can learn from human experience, which can make the model more accurate.

## 1. Introduction

Human action recognition is currently a popular research field. However, it is a complex task, involving recognition of the action performed by a person, interaction between people, and interaction between people and the environment. With the improvement in the living standards of people, the demand for action recognition is increasing. Therefore, research on action recognition has gained importance. For example, in terms of human–computer interaction, machines need to accurately recognize the actions of humans and respond appropriately to assist humans. An effective action recognition method will make people feel more comfortable. Action recognition is of immense use in many fields. In video surveillance, action recognition can detect a shoplifter in a supermarket and dial the police. It can detect people requiring assistance on the road, such as a senior citizen who has accidently fallen on the road, and seek timely aid. During autonomous driving, action recognition can recognize the activity of a passerby to determine whether the person’s walking trajectory will interfere with the current driving path so that it can be adjusted in time.

Currently, there are two methods for action recognition. One is the traditional method, which requires artificial features. The other is a method based on deep learning. The traditional method requires the researcher to define the features of the actions, and train the corresponding classifier accordingly. Then, the researcher will use the trained classifier to generate predictions. Traditional action recognition methods have been developed over many years and have several advantages. Compared with the deep learning method, the traditional method is easy to understand, the features can be modified artificially, and the calculation speed is faster. However, it has some shortcomings as well. The quality of the feature design has a significant effect on the accuracy of recognition. Moreover, the accuracy of recognition is not very high. The deep learning method does not need to manually define the features; it can automatically learn the features with the data, which reduces the influence of human factors and improves the recognition accuracy. However, certain special features can still be manually extracted and put into the model. With the development of deep learning and GPUs, the mainstream methods for action recognition have gradually changed from traditional methods to deep learning methods. Moreover, deep learning methods have achieved good performance on many datasets. The features in the action recognition methods based on deep learning are automatically generated according to the training data, i.e., these methods extract key features from the data. Therefore, the recognition accuracy is greatly affected by the data. A large amount of data should be used to improve the accuracy of the model.

Presently, the datasets used for action recognition are increasing in size, resulting in increasing labor cost for collecting and labeling videos. From the KTH (six categories, 2391 videos) to the current Kinetics-700 (700 categories, approximately 650,000 videos), the number and categories of videos have increased exponentially. Increasing the size of the dataset will result in increased labor cost to annotate the videos. A very large dataset will require considerable time for training, and the addition of a new class will require retraining the model. In this case, the training cost will be very high. These are some of the current challenges in action recognition. However, in contrast, human beings do not need a large amount of data for action recognition. They can learn how to recognize new objects with few data or only text descriptions.

In order to solve the problems of excessive training data and labor cost, researchers have attempted to combine action recognition methods and zero-shot learning (ZSL). In 2009, zero-shot was applied for the first time to process images [1]. This method can recognize the picture of a new class, even if it has not been trained with this class before. Therefore, this method is convenient when adding a new class, which has made it an attractive option for researchers and inspired new research goals. ZSL can imitate humans and use knowledge that has been learned in the past to reason unseen images.

We use ZSL in action recognition, called zero-shot action recognition (ZSAR). With ZSAR, we no longer need a large number of labeled videos as training datasets. We only need to know the features of an action, such as the speed of performing the action and whether it is performed indoors or outdoors. Then, we determine if a video involves this action based on these features. ZSAR does not involve high labor costs, but it is very dependent on the selection of features. Despite the use of deep learning, we rarely need to extract features manually. However, for ZSL, it is necessary to select the appropriate features artificially. Failure to select good features will result in poor accuracy. Therefore, the extraction of good features is an important research focus for ZSL.

The two-stream graph convolutional network (TS-GCN) [2] is a high-accuracy model that builds knowledge graphs of actions and objects to predict unseen videos on the basis of objects in the video. However, this model only uses the RGB data of the videos and does not use human motion data at all. This approach ignores a large amount of useful data. The addition of motion data to the model can improve the model’s accuracy. In this study, we added a branch to the baseline model, which has two branches, to improve the model’s accuracy. The added stream is designed for processing motion data. We used a pre-trained spatial temporal graph convolutional network (ST-GCN) [3] to extract the motion data of the video and then used the deep visual-semantic embedding model (DeViSE) [4] to predict the unseen video. This approach compensates for the shortcomings of the baseline model and improves model accuracy.

The original input data of our model has two parts, one is RGB image data, the other is human skeleton data. The RGB data is captured with cameras. The image sensor of the camera converts the optical information into the digital image data and saves it into the video. For skeleton data, the traditional method is to wear sensors to capture the motion of human joints. However, this method has limitations and is not convenient to use in some cases, such as outdoors or underwater. If we want to detect the movement of athletes in the competition, we have to put on sensors for everyone to compete, but it is difficult to do. Since the cost of this method is high, we need to buy special equipment to capture the motion of human joints. Therefore in this paper, we use a special module, such as OpenPose [5], to recognize the skeleton data from images. This method is cheap and convenient.

The major contributions of this study are as follows.

- We propose a three-stream graph convolutional network (three-stream GCN) model. This model can process not only the RGB data of the video, but also the motion data of the person. With the motion branch, we can extract the motion data of the video and recognize the unseen video;
- By comparing with the accuracy of the baseline model, we evaluate the effectiveness of our method. In addition, we evaluate the effects of several parameters;
- We experimentally prove that our model can learn from human experience. We generate a training set based on human experience and improve the accuracy of the model.

An earlier version of this paper was presented at an international conference [6]. In this extended version, we have used additional datasets and methods to evaluate our model. The number of datasets has been increased from one to three, and our proposed method is now compared with four methods, including the baseline method. Moreover, we use three word embeddings in the motion branch instead of one. In addition, we calculate the distribution of the optimal weight and visualize the semantic space. To improve the comprehensibility of the paper, we have revised the description of the proposed method and its evaluation.

## 2. Related Work

### 2.1. Action Recognition

With the development of action recognition in the last few years, researchers have been studying how to extract video features so that a machine can better classify the video. Several models are highly popular, such as two-stream networks [7], 3D convolutional neural networks (C3D) [8], and two-stream inflated 3D ConvNet (I3D) [9]. Two-stream networks use convolutional neural networks (CNNs) to process the RGB images and optical flow data of the video, and then place them together at the end. C3D treats video as a 3D image and uses a CNN with a 3D convolution kernel to process videos. These models all use the RGB data of the video to recognize actions.

Human skeleton data can also be used. Compared with RGB images, the skeleton has the advantage of having clear and simple features and it is not easily affected by the appearance. In contrast, the method based on RGB images is easily disturbed by changes in appearance, such as changes in brightness or different skin colors in the training set and testing set, which affect the accuracy of recognition. The input of a skeleton-based model is the position of human joints; as a result, the model is hardly affected by changes in appearance. If the skeleton data are not included in the dataset, OpenPose [5] must first be used to extract the skeleton data from the RGB images. Early skeleton-based action recognition methods [10,11,12,13,14,15,16] rarely considered the relationship between joints. These methods use simple models such as support vector machine (SVM) or fully connected networks. Yan proposed ST-GCN [3], which uses GCN to process the skeleton data of humans. Recently, GCN has been increasingly used in models. The common method to judge the relationship between two joints is based on whether they are connected by bones. The work of [17] proposed the spatio-temporal graph routing (STGR) scheme, which can reveal the potential connection between two joints even if they are not directly connected. Su [18] proposed a model based on an encoder–decoder recurrent neural network for action recognition. The input of this model is skeleton data. Khalid [19] proposed a multi-modal three-stream network for action recognition. This model used two-stream networks to process RGB data and a CNN to process skeleton data.

### 2.2. Zero-Shot Learning

In ZSL, because the training data and test data are separate, there is no commonality between them. Therefore, we need to establish a connection between them. We can then obtain the relevant knowledge from the seen data and use this knowledge to predict the unseen data. The output of the traditional image classification model is a one-hot vector, but this method cannot recognize unseen data. Therefore, we need to add a new space between the input and output, called the shared semantic space. In this case, the labels of each category are no longer independent, but are represented by vectors in the shared semantic space. The ZSL model has three spaces: (1) Visual space, composed of all images or videos; (2) label space, composed of the labels of all categories; and (3) shared semantic space, obtained through artificial definition (attribute space) or learning by using existing knowledge (word embedding space).

The training of ZSL involves the creation of a mapping model from the visual space to the shared semantic space. After the image data is mapped, it will reach the shared semantic space. In an ideal situation, the distance between the data of the same category will be small in this space. The input data are divided into seen and unseen data. The seen data constitute the training dataset, which contains the complete image data and label information. The unseen data constitute the test dataset, which has neither image data nor category information. We only know the names of the labels. In the input part of the model, there is a model that extracts features from the images. There are two methods to extract the features. One is the traditional method, which uses artificially created features such as HSV color histogram, SIFT, or SURF. The other is a deep learning model based on CNN, such as GoogLeNet, ResNet, or VGG, which is currently the mainstream method. Next, we use a mapping model to map the features of the image to the shared semantic space. The commonly used shared semantic space can be the artificially defined features or the word embedding space of natural language processing (NLP). The shared semantic space builds a bridge from the low-level visual space to the high-level label space. In this space, the seen and unseen classes can share knowledge. In other words, during training, the model uses the seen data to construct the shared semantic space and then, during testing, it can use the knowledge of this space to classify the unseen data.

At the same time, we need to map the category information of seen and unseen data to the shared semantic space. If the artificially defined features are used as the shared semantic space, the value of each category should be manually set in the shared semantic space. If the word embedding space is chosen, the name of each category should be mapped to this space. In the training step, we mainly use the seen data to train the mapping model from the image to the semantic space. After the completion of training, when an instance of unseen data is presented, the model will output the corresponding vector, which represents the position of this data in the shared semantic space. We compare this vector with the values of all categories in the shared semantic space, and select the closest category as the label of this data. The nearest neighbor method is usually used to find the nearest label. Thus, an unseen instance of data can be classified with the ZSL method.

**Figure 1:**
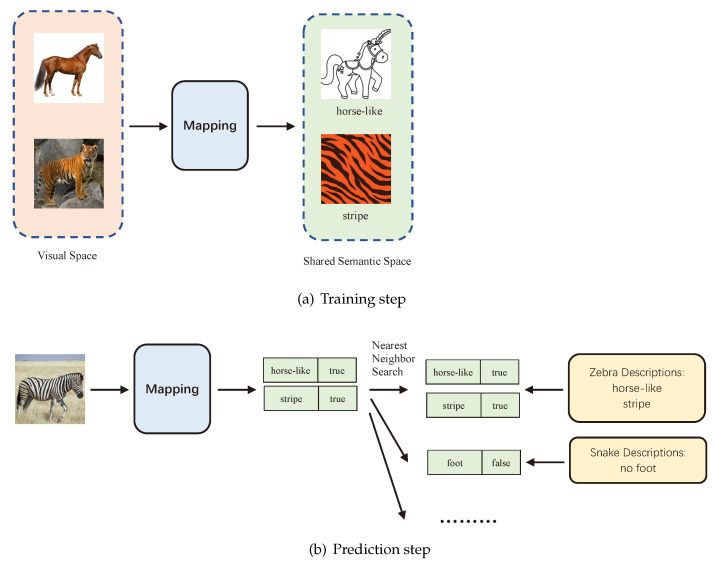
Zero-shot learning.

Figure 1a depicts the training process in ZSL. The inputs of the mapping model are images of horses and tigers, and the outputs of the model are the features of horses and tigers. The feature of a horse is horse-like, and the feature of a tiger is its stripe. After training the model with the training dataset, the model can recognize whether there are horse-like or stripe features on the images. Figure 1b is the prediction process. We first use the text data to generate the features of all unseen animals. Then, we input an unknown image into the mapping model. The mapping model can generate the features of the animal in this image. Finally, we use the nearest neighbor search algorithm to search in the features of all unseen animals. The vector closest to the current image feature is identified, and the label corresponding to this vector is used as the label of this unknown image.

Larochelle proposed ZSL in 2008 to solve the problem of character recognition [20]. Lampert applied ZSL to the field of computer vision. His model can classify unseen animals. The AwA2 dataset [1] was assembled to evaluate the algorithm for ZSL. Thereafter, ZSL has been extensively researched. Later, Akata proposed label-embedding [21] and structured embedding [22]. Early ZSL methods [23,24,25] used attributes to represent the categories. Some researchers have used generative adversarial networks (GANs) to solve the zero-shot issue. The methods reported in [26,27,28,29] use GAN to generate the corresponding images based on the features of the category. Recently, researchers have used word embedding to generate the shared semantic space. DeViSE is a classic model in ZSL. It can learn the projections from the visual features to the embedding of labels. The model can then use a nearest neighbor search to find the label of the unseen video. Other methods to solve ZSL include error correcting codes [30] and generative models [31].

### 2.3. Zero-Shot Action Recognition

In order to solve the problem of very large datasets for action recognition, we combine action recognition with ZSL, which is called zero-shot action recognition (ZSAR). For example, consider a case where the training dataset has walking videos, but the model needs to recognize a running video, which is an instance of unseen data. We know that running and walking are very similar actions, but the action of running is faster than that of walking. Based on this feature, the model can recognize the running video, even if it is unfamiliar.

ZSAR and ZSL are very similar, and the only difference is in the feature extraction model. We replaced the image processing model with the video processing model. To recognize an action based on the appearance information contained in the videos, we can select a model that can handle RGB data. The iDT [32] model is a traditional video feature extraction model. Models based on deep learning include C3D [8], TSN [33], and I3D [9]. If the recognition is based on human skeleton data, ST-GCN [3] can be selected to extract the features. Current ZSAR research is focused mainly on finding the projections from the visual features to the semantic space, so that similar classes can be brought closer in the semantic space.

Xu [34] used semantic embedding space to identify unseen actions. Jain proposed objects2action [35] to recognize actions. This method does not require video data; it only needs to detect the objects on the video, and then infer the action based on the relationship between these objects and the action. Jasani and Mazagonwalla [36] built a prediction model, but its accuracy is superior to that of other models only when the relationship between the seen and unseen data is far. TS-GCN [2] uses a knowledge graph to learn the relationships between objects and actions and achieves high accuracy. However, this model uses only the video RGB image without the human motion data. In this study, we selected this model as the baseline.

Although ZSL has made significant progress, there has been limited research on action recognition based on ZSL. Moreover, most of the methods simply apply the method of static image classification to action recognition. These methods use static images of the video, and few models can process the time series data of the video. Image-based recognition methods are strongly affected by the light and background; these issues need to be resolved.

## 3. Method

**Figure 2:**
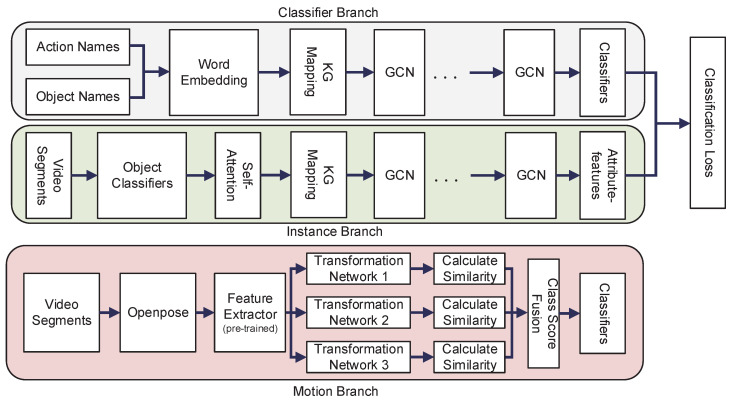
Three-stream graph convolutional network architecture.

Our model consists of two components: (1) TS-GCN, which has two branches, and (2) our proposed motion branch. We add this branch to the original model to process human motion data. Figure 2 shows the architecture of our model.

### 3.1. Two-Stream Graph Convolutional Networks

Many recent ZSAR models, including TS-GCN, use knowledge graphs. The knowledge graph contains the relationship between words. TS-GCN uses a knowledge graph to calculate the relationship between objects and actions. When the model knows the objects in the video, it can predict the label of an unseen video with the relationship between objects and actions. This model has two branches: A classifier branch and an instance branch.

The Classifier Branch has a GCN. The nodes of its graph are composed of objects and actions. If the relationship between an object and action is close, an edge exists between them. The input of the classifier branch word embedding vectors is converted from the names of all objects and actions. The word embeddings are converted by Global Vectors (GloVe) [37]. Each word is converted to a vector. If a name has multiple words, we add the vectors of these words together. If a word is not in GloVe, then we manually replace it with a synonym. Finally, we put all word embeddings into GCN as input.

The Instance Branch is composed of object classifiers and GCN. We divide the video into 16 segments of equal length, and put each frame of each segment into GoogLeNet [38] to identify the objects. We employ a GoogLeNet model trained on a 12,988-category shuffle [39]. The input of GoogLeNet is a frame, and the output is a 1 × 12,988 vector, which is used as the object score. We put this vector into a self-attention module, and then use the output of the module as the input of the GCN.

We use the information in ConceptNet [38] to build the GCN and calculate the relatedness between each action and all objects. The top 100 most relevant objects are selected for each action. If a word is not in ConceptNet, we will manually replace it with a synonym. Unlike the classifier branch, this GCN uses all objects as nodes without actions.

### 3.2. Three-Stream Graph Convolutional Networks

DeViSE: This is a common ZSAR method that projects the name of each label to a vector by word embedding. Words with similar meanings also have similar positions in the vector space. In this way, if we put the seen video and the word embedding of its label into the model, then we can determine the projection from video to word embedding. After training, we put the unseen video into the model to predict the word embedding of the video. Finally, with a nearest neighbor search, we can obtain the label closest to the current vector as the final result.

Multiple word embeddings: If we use different word embeddings to process the same word, the resulting vector will be different. Therefore, the result is affected by the word embedding. If we have chosen an inappropriate word embedding, the result of the model will be very poor. For example, GoogleNews is trained with the text reported in news. If informal words are used, the result may not be accurate. Hence, when processing informal words in our data, the GoogleNews model can be used to convert the words into vectors, but the results may not be accurate. In order to reduce this effect and improve the stability of the model, we use three different word embeddings separately. Then, their results are combined to generate the final result.

Motion branch: We first use OpenPose or another model to extract the human skeleton data in the video to generate 2D or 3D coordinate sequences. We then put these coordinate data into the model. The model can recognize actions with human joint information. For the ST-GCN model, we use the kinetics [40] for pre-training. After pre-training, we remove the last layer of the model and use its output as the feature of the video. The feature of the video is a 1 × 256 vector. We employ the DeViSE model and use the seen video as a training dataset to train the model. The DeViSE model can learn the projection from video features to word embeddings. According to [41], we use these three models to map the feature data of the videos to three shared semantic spaces separately. We employ ConceptNet Numberbatch, GloVe, and GoogleNews to obtain the word embeddings. After training, we can input the unseen video into the model and predict its word embeddings. Then, we calculate the similarity between the mapped semantic vectors and the word embedding of each action label as the score of each action. Finally, we calculate the average of the output of the three branches as the final score.

Three-stream graph convolutional networks: The first two branches are used as one model, and finally a 1 × 101 vector is predicted. This vector represents the possibility of the label of the video. The third branch also predicts the possibility of the label of the video. A larger value means a greater probability that the video is the label. We train the two models separately. After training, we use these two models to predict unseen videos separately. As an end-to-end model, the output of two-stream GCN is the probability of each class. We take this probability as $score_{1}$. For the motion branch, we convert all labels to word embedding. We put the features of skeleton into the model, and then we get the predicted word embedding, which is a vector. Then we calculate the cosine similarity between this predicted word embedding and word embeddings of all labels:

(1) $$s\left(p,q_{c}\right)=\frac{<p,q_{c}>}{∥p∥∥q_{c}∥},$$

where *p* is the predicted word embedding and $q_{c}$ is the word embedding of class *c*, c=1,2,…. They are any two vectors of the same dimension. $<p,q_{c}>$ is the inner product. According to Equation (1), we can calculate the possibilities of all classes. Then we concatenate all the possibilities as $score_{2}$. We finally calculate the weighted sum of the predicted scores as the final prediction result:

(2) $$score=score_{1}+score_{2}\times w,$$

where $score_{1}$ and $score_{2}$ are the scores of the baseline and motion branch, respectively. *w* is the weight of the motion branch.

### 3.3. Knowledge Graph

A knowledge graph describes knowledge using visualization methods, which can show the relationship between objects. The knowledge graph was proposed by Google and was first applied in the search engine. For example, when we search for “apple” with Google, the results include not only the fruit, but also Apple Inc. (Cupertino, United States), mobile phones, and computers, which are all related to this word. In this study, ConceptNet was used as the knowledge graph. The nodes of ConceptNet are the words in various languages, and the edges are the relationships between the words. It contains over 21 million edges, over 8 million nodes, and 83 languages. ConceptNet has approximately 1.5 million English words. In ZSAR, we use the knowledge graph to show the relationship between objects and actions, and then recognize the action based on the objects that appear in the video. For example, the presence of food in a video could indicate the action of eating or cooking. Furthermore, if a pot does not appear in the video, then this action is likely to be that of eating.

### 3.4. Extracting Features

Extracting reliable features is very important in ZSAR, as it affects the accuracy of the result. ZSAR uses three methods for extracting the features: (1) Manual extraction of features [1]. This method requires high labor cost, as the features of each action should be analyzed and annotated. The accuracy of annotation will be low in the case of features that people cannot easily distinguish, such as variable motion, as precise features cannot be extracted. (2) Extraction of the information of actions from the text and generation of features [4]. One of the common methods is to use a large number of text data on the Internet to create a model, and convert each action name to word embedding with this model. The word embedding is the feature of this action. (3) Generation of features of actions using the information of knowledge graphs [2]. In ZSAR, people can recognize the relationship between actions and objects by building knowledge graphs. By using these relationships, we can predict the label of a video according to the objects appearing in it, even in the case of an unseen action. Method 1 uses several features of the actions, which has very little information. Method 2 converts each word into a vector that represents the position of the word in the vector space. Then, this data is processed using fully connected layers (FCNs). Method 3 uses knowledge graphs, which can be processed by a GCN. In comparison with FCNs, a GCN can process not only the data of nodes, but also the relationship between the nodes. Method 3 can process more data than method 2, therefore, it has more potential.

### 3.5. Object Detection

There are several object detection methods, such as YOLO, SSD, and R-CNN. The input of the object detection model is a picture, and the outputs are the label and location of each object. These methods provide extensive information about the objects. However, the presence of too many categories in the dataset will considerably increase the difficulty of detection, resulting in a decline in the accuracy. In this paper, in order to improve the accuracy of action recognition, we need to use several objects to predict the action. If the categories of the objects are few, less information will be available for use, which will reduce the accuracy. Our model only uses the information about the relationship between the objects and actions, and cannot use the location information of the object. Therefore, we use the image classification model to detect the object. The input of the image classification model is an image, and the output is only the label, without the location information. Most importantly, the number of classes in the model can be very high, resulting in high accuracy. The label with the highest probability, N, is the output as the object that appears on this image.

## 4. Experiments

In this section, we discuss the experiments for performance evaluation of the baseline and our model, conducted on UCF101 [42].

### 4.1. Dataset

Datasets and splits: We used UCF101, HMDB51, and Olympic Sports as the datasets. UCF101 is a video dataset with 101 categories and a total of 13,320 videos. We randomly divided it into 51 seen videos for training and 50 unseen videos for testing. HMDB51 contains 51 categories and 6849 videos. We randomly divided it into 26 seen videos for training and 25 unseen videos for testing. Olympic Sports contains 150 videos on sports, with a total of 13 actions. We randomly divided it into 7 seen videos for training and 6 unseen videos. We used Numberbatch (9,161,912 words), GloVe (2,196,017 words), and GoogleNews (3 billion words) as the word embeddings. The dimensions of their words are all 300.

### 4.2. Baseline Comparison

Two-stream graph convolutional networks: In the construction of GCN, we selected the top 100 most relevant objects of each action and removed the irrelevant objects. Of the original 12,988 objects, 3467 remained, and we put them together to generate the graph of the instance branch. Then, we added the actions into the graph, and generated the graph of the classifier branch. After building the model, we put the word embeddings of actions and objects into the classifier branch, and put the video into the instance branch. The output of the model is a 1 × 101 vector, which represents the action probabilities. We selected the most likely action as the final result. From this result, we calculated the accuracy of this model.

Three-stream graph convolutional networks: We employed a TS-GCN model pre-trained using kinetics. We put the skeleton of the seen video into the model to extract features. Then, we put the extracted features into a three-layer network, with layer sizes of 256, 1000, and 300. We trained it for 50 epochs with SGD, a learning rate of 0.2, dropout of 0.5, and batch size of 64.

**Figure 3:**
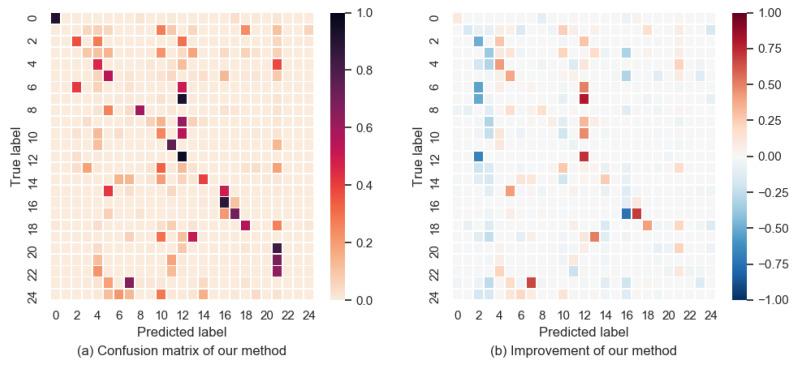
Confusion matrix of our model (UCF101).

After training the model using the seen video, we input the unseen video into the model for prediction. After the prediction of the motion branch, we combine the two models together with the weight of 0.1. We then obtain the final prediction result, as shown in Figure 3. Table 1 shows the accuracy of each model. Figure 3a depicts the confusion matrix of our model. Figure 3b shows the improvement from the baseline to our model:

(3) $$V=V_{2}-V_{1},$$

where $V_{2}$ and $V_{1}$ are the confusion matrices of our model and the baseline. Figure 3b shows that there are more red squares on the diagonal than blue, which means that the accuracy of our model is higher than that of the baseline. The results may be different in parameter settings or some details. The accuracy of the baseline is lower than that previously reported [2]. However, it can be seen from the comparison that the motion branch improves the accuracy of the baseline.

**Figure 4:**
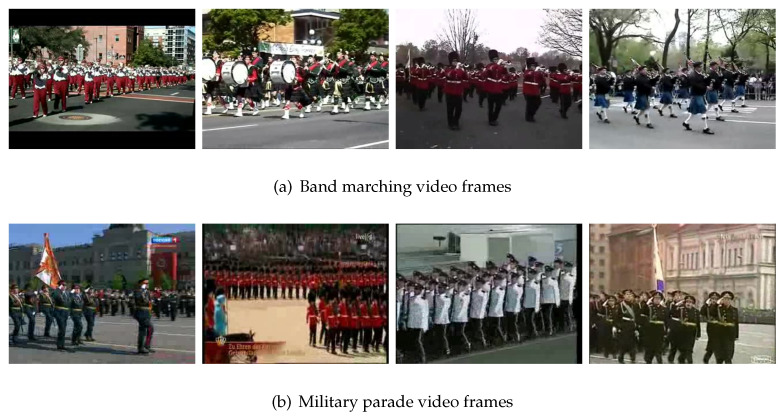
Mistake-prone video frames.

In Figure 3a, we can see that some classes have very low accuracy, such as band marching. Half of its videos are predicted as a military parade. Figure 4 shows the video frames for band marching and a military parade. From this figure, we can see that the two classes of videos are very similar, in terms of not only the RGB images but also the human motions, thus resulting in low accuracy. In fact, the difference between these two classes of actions is that the person in the band marching video plays an instrument while walking.

**Figure 5:**
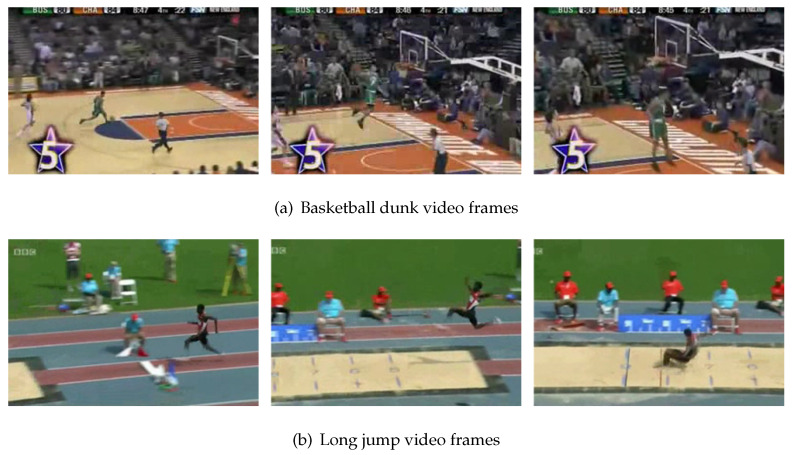
Video frames for which the performance worsens upon adding the motion branch.

After analyzing Figure 3b, we find that the result of label 2 has worsened whereas that of label 12 has improved. Label 2 is basketball dunking and label 12 is long jumping. The baseline classified most of these two classes of videos as basketball dunking. After adding the motion branch, most of these videos were classified as long jumping. Figure 5 shows the example images of basketball dunking and long jumping. The people in the video run and then jump. These two classes of actions are very similar and thus are easily misclassified.

### 4.3. Learning from Human Experience

In order to improve the accuracy of the model, we need to find which category has a low accuracy, and analyze why it is low. For two-stream GCN, we firstly calculate the accuracy of each class, and sort them from small to large and calculate what each class is most often wrongly predicted. Then we find the category which have potential to improve the accuracy. If an object exists in the correct category but not in the wrong category, we can improve the accuracy by modifying the relationship between object and action. By analyzing the experimental results, we find that the accuracy of band marching is 0%. Most of the videos are classified as a military parade. From Figure 4, we can see that the two classes of videos are very similar. The difference between these two classes of actions is that the person in the band marching video plays an instrument while walking. Therefore, we need to improve the relationship between instruments and band marching as well as reduce the relationship between instruments and military parade. This will improve the accuracy of the band marching.

We generate the training data of the two-stream GCN, and the input is the score of each object. Then, we use GloVe to convert each object into word embedding and calculate the similarity between it and the instrument. We use objects with a similarity of more than 0.3 as instruments. Several instruments are randomly selected, the score is set to a decimal greater than 0.2, and these scores are used as input. There are two kinds of output data. One is the word embedding of band marching. We convert the military parade to the opposite vector of word embedding. This vector is another output that can reduce the possibility of it being a military parade. We randomly generate 120 sets of data. The amount of data is set according to experience. Too much data will affect the accuracy of other categories, whereas too little data may not improve the model. Next, the training set is used to train the model and the generated data is used to continue training. We use the test set to evaluate the new model; the results are shown in Table 2. We can see that our method improves the recognition ability of the model for band marching and does not affect other categories. However, for all categories, the accuracy only increased by 0.2%, which is too small. This is because we have only modified one class. The more rules that are added, the higher the accuracy that can be obtained.

As for the motion branch, according to human experience, the lower body of band marching is walking and the upper body is playing instruments. Furthermore, the number of people is very large and the team is neat. According to these rules, we can use the skeleton data of walking and playing instruments to make the data for band marching. Using this method, we can improve the accuracy of motion branch for band marching. Due to the complexity of this method, we plan to evaluate its performance in future in a separate study.

### 4.4. Different Word Embeddings

To evaluate the effects of different word embeddings of the model, we used two different word embeddings, and then calculated the ZSAR accuracy. We used Numberbatch (9,161,912 words), GloVe (2,196,017 words), and GoogleNews (3 billion words) as the word embeddings. The dimensions of their words are all 300.

As shown in Table 3, the weight and accuracy have different relationships in different word embeddings. “All” denotes that the three word embeddings are used together. From the table, it is apparent that the model is sensitive to the word embeddings. The accuracies of different word embeddings are also different. Therefore, this model is sensitive to the word embedding method. We use the three word embeddings together to reduce the effect of this sensitivity.

### 4.5. Different Weights

**Figure 6:**
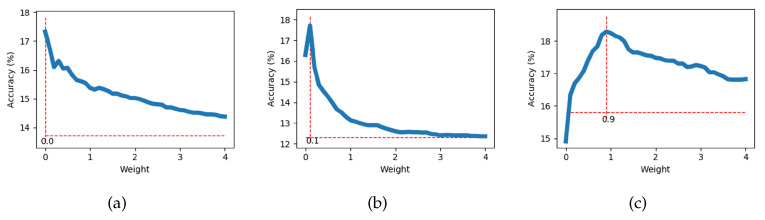
Changes in accuracy with different weights.

**Figure 7:**
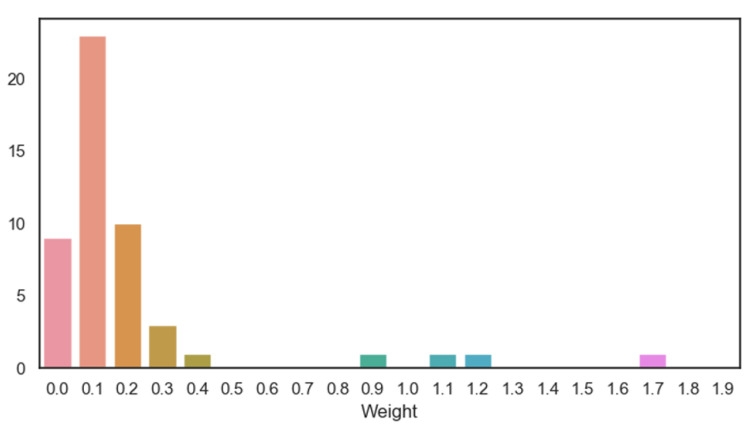
Number of optimal *w* in 50 models.

We randomly generate 50 different methods to split the UCF101 dataset, and use these datasets to train 50 models. The dataset is divided into two parts: 51 seen classes and 50 unseen classes. We calculate the optimal weight that can yield the highest accuracy of the model. Finally, we obtain 50 optimal weights. The calculation method is as follows. According to Equation (2), we select w=0.1,0.2,0.3…. Then, we use *w* to calculate the accuracy of the model. When w=0, there is no motion branch but only the output of the baseline model. After processing all values of *w*, we select the model with the highest accuracy and record the corresponding value of *w*. Figure 6 shows the curves of the relationship between *w* and the accuracy when training is performed with three datasets. We can see that for different datasets, the optimal value of *w* is different. The addition of a motion branch can improve the accuracy of the model. The optimal *w* of 50 models is counted and the results are depicted in Figure 7. The horizontal axis denotes the value of the optimal *w*, and the vertical axis denotes the number of occurrences of this value among the 50 optimal *w*. From the figure, we can see that the value with the largest number is 0.1, i.e., when w=0.1, the model is more likely to have the highest accuracy.

### 4.6. Visualization of Semantic Space with Different Word Embeddings

**Figure 8:**
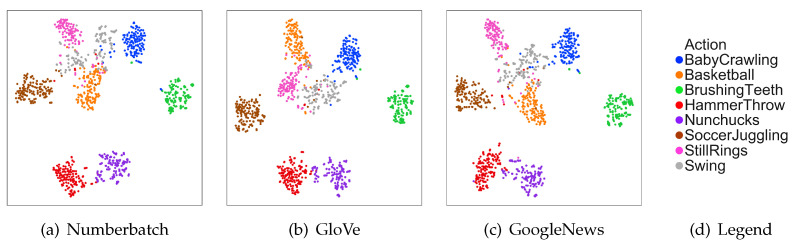
Visualization of semantic space with different word embeddings.

To observe the effect of different word embedding on the accuracy more intuitively, we reduced the number of transformation networks of the motion branch from 3 to 1. We projected all the videos into different semantic spaces with the motion branch and then visualized them with t-SNE [43]. The result is shown in Figure 8. We can see that the results of mapping the videos to different word embeddings are similar. However, there will be some differences at the boundary of two categories. Each word embedding has its own advantages. Hence, we need to use the three word embeddings together to make the model more stable rather than attempt to choose the best word embedding.

## 5. Conclusions

In this study, first we proposed a three-stream GCN model for ZSAR. The proposed model not only analyzed the data of the objects in a video, but also added the data of human motion. We added a stream to the original model. Experiments to compare the accuracy of our model with that of the baseline established the effectiveness of our method. Second, we added a new experiment to prove that our model could learn from human experience. We generated training data based on human experience and improved the accuracy of the model. Moreover, we modified the weight of the model and tested the effect of weight change on the accuracy. We also evaluated the effect of different word embeddings on the accuracy.

This method uses both RGB data and motion data, which improves the accuracy of recognition. However, it uses the outputs of two models. The two parts of the model process the data separately. This prevents the model from making full use of the relationship between the RGB data and motion data, which needs to be addressed. In future, we will combine them such that the model can fully learn the relationship between them. We can incorporate additional features to the model, such as the location data of the objects and the audio data from the video. These enhancements will further improve recognition accuracy.

## Figures and Tables

**Table 1:** Accuracy of each model.

Model	Reference	UCF101	HMDB51	Olympic Sports
DAP	CVPR2009	14.3	N/A	43.2
IAP	CVPR2009	12.8	N/A	42.7
ESZSL	ICML2015	14.5	17.6	37.9
Two-Stream GCN (baseline)	AAAI2019	17.0	19.3	51.3
Motion Branch	Ours	14.8	16.6	46.9
Three-Stream GCN	Ours	17.9	20.4	54.1

**Table 2:** Accuracy before and after learning from human experience.

Categories	Before (%)	After (%)
Band Marching	0	14.8
All Categories	17.0	17.2

**Table 3:** Accuracy of different word embeddings.

Word Embedding	Motion Branch Accuracy (%)	Three-Stream Accuracy (%)
Numberbatch	13.7	15.6
GloVe.840B.300d	13.2	17.0
GoogleNews	12.9	16.1
All	14.8	17.9

## Data Availability

The data presented in this study are available on request from the corresponding author.

## References

[1] Lampert C.H., Nickisch H., Harmeling S. Learning to detect unseen object classes by between-class attribute transfer. Proceedings of the 2009 IEEE Conference on Computer Vision and Pattern Recognition.

[2] Gao J., Zhang T., Xu C. I know the relationships: Zero-shot action recognition via two-stream graph convolutional networks and knowledge graphs. Proceedings of the AAAI Conference on Artificial Intelligence.

[3] Yan S., Xiong Y., Lin D. Spatial temporal graph convolutional networks for skeleton-based action recognition. Proceedings of the Thirty-Second AAAI Conference on Artificial Intelligence.

[4] Frome A., Corrado G.S., Shlens J., Bengio S., Dean J., Ranzato M., Mikolov T. Devise: A deep visual-semantic embedding model. Proceedings of the Advances in Neural Information Processing Systems.

[5] Cao Z., Hidalgo Martinez G., Simon T., Wei S., Sheikh Y.A. (2019). OpenPose: Realtime Multi-Person 2D Pose Estimation using Part Affinity Fields. IEEE Trans. Pattern Anal. Mach. Intell..

[6] Wu N., Kawamoto K. Three-Stream Graph Convolutional Networks for Zero-Shot Action Recognition. Proceedings of the 2020 Joint 11th International Conference on Soft Computing and Intelligent Systems and 21st International Symposium on Advanced Intelligent Systems (SCIS-ISIS).

[7] Simonyan K., Zisserman A. Two-stream convolutional networks for action recognition in videos. Proceedings of the Advances in Neural Information Processing Systems.

[8] Ji S., Xu W., Yang M., Yu K. (2012). 3D convolutional neural networks for human action recognition. IEEE Trans. Pattern Anal. Mach. Intell..

[9] Carreira J., Zisserman A. Quo vadis, action recognition? A new model and the kinetics dataset. Proceedings of the IEEE Conference on Computer Vision and Pattern Recognition.

[10] Si C., Jing Y., Wang W., Wang L., Tan T. Skeleton-based action recognition with spatial reasoning and temporal stack learning. Proceedings of the European Conference on Computer Vision (ECCV).

[11] Wang J., Liu Z., Wu Y., Yuan J. Mining actionlet ensemble for action recognition with depth cameras. Proceedings of the 2012 IEEE Conference on Computer Vision and Pattern Recognition.

[12] Vemulapalli R., Arrate F., Chellappa R. Human action recognition by representing 3d skeletons as points in a lie group. Proceedings of the IEEE Conference on Computer Vision and Pattern Recognition.

[13] Du Y., Wang W., Wang L. Hierarchical recurrent neural network for skeleton based action recognition. Proceedings of the IEEE Conference on Computer Vision and Pattern Recognition.

[14] Song S., Lan C., Xing J., Zeng W., Liu J. An end-to-end spatio-temporal attention model for human action recognition from skeleton data. Proceedings of the AAAI Conference on Artificial Intelligence.

[15] Zhang P., Lan C., Xing J., Zeng W., Xue J., Zheng N. View adaptive recurrent neural networks for high performance human action recognition from skeleton data. Proceedings of the IEEE International Conference on Computer Vision.

[16] Xie C., Li C., Zhang B., Chen C., Han J., Zou C., Liu J. (2018). Memory attention networks for skeleton-based action recognition. arXiv.

[17] Li B., Li X., Zhang Z., Wu F. Spatio-temporal graph routing for skeleton-based action recognition. Proceedings of the AAAI Conference on Artificial Intelligence.

[18] Su K., Liu X., Shlizerman E. Predict & cluster: Unsupervised skeleton based action recognition. Proceedings of the IEEE/CVF Conference on Computer Vision and Pattern Recognition.

[19] Khalid M.U., Yu J. Multi-modal three-stream network for action recognition. Proceedings of the 2018 24th International Conference on Pattern Recognition (ICPR).

[20] Larochelle H., Erhan D., Bengio Y. (2008). Zero-data learning of new tasks. AAAI.

[21] Akata Z., Perronnin F., Harchaoui Z., Schmid C. Label-embedding for attribute-based classification. Proceedings of the IEEE Conference on Computer Vision and Pattern Recognition.

[22] Akata Z., Lee H., Schiele B. (2014). Zero-shot learning with structured embeddings. arXiv.

[23] Cheng Y., Qiao X., Wang X., Yu Q. (2017). Random forest classifier for zero-shot learning based on relative attribute. IEEE Trans. Neural Netw. Learn. Syst..

[24] Demirel B., Gokberk Cinbis R., Ikizler-Cinbis N. Attributes2classname: A discriminative model for attribute-based unsupervised zero-shot learning. Proceedings of the IEEE International Conference on Computer Vision.

[25] Fu Y., Hospedales T.M., Xiang T., Gong S. (2015). Transductive multi-view zero-shot learning. IEEE Trans. Pattern Anal. Mach. Intell..

[26] Zhang C., Peng Y. (2018). Visual data synthesis via gan for zero-shot video classification. arXiv.

[27] Xian Y., Lorenz T., Schiele B., Akata Z. Feature generating networks for zero-shot learning. Proceedings of the IEEE Conference on Computer Vision and Pattern Recognition.

[28] Sariyildiz M.B., Cinbis R.G. Gradient matching generative networks for zero-shot learning. Proceedings of the IEEE/CVF Conference on Computer Vision and Pattern Recognition.

[29] Ji Z., Chen K., Wang J., Yu Y., Zhang Z. (2020). Multi-modal generative adversarial network for zero-shot learning. Knowl. Based Syst..

[30] Qin J., Liu L., Shao L., Shen F., Ni B., Chen J., Wang Y. Zero-shot action recognition with error-correcting output codes. Proceedings of the IEEE Conference on Computer Vision and Pattern Recognition.

[31] Mishra A., Verma V.K., Reddy M.S.K., Arulkumar S., Rai P., Mittal A. A generative approach to zero-shot and few-shot action recognition. Proceedings of the 2018 IEEE Winter Conference on Applications of Computer Vision (WACV).

[32] Wang H., Schmid C. Action recognition with improved trajectories. Proceedings of the IEEE International Conference on Computer Vision.

[33] Wang L., Xiong Y., Wang Z., Qiao Y., Lin D., Tang X., Van Gool L. (2016). Temporal segment networks: Towards good practices for deep action recognition. European Conference on Computer Vision.

[34] Xu X., Hospedales T., Gong S. Semantic embedding space for zero-shot action recognition. Proceedings of the 2015 IEEE International Conference on Image Processing (ICIP).

[35] Jain M., Van Gemert J.C., Mensink T., Snoek C.G. Objects2action: Classifying and localizing actions without any video example. Proceedings of the IEEE International Conference on Computer Vision.

[36] Jasani B., Mazagonwalla A. (2019). Skeleton based zero shot action recognition in joint pose-language semantic space. arXiv.

[37] Pennington J., Socher R., Manning C.D. Glove: Global vectors for word representation. Proceedings of the 2014 Conference on Empirical Methods in Natural Language Processing (EMNLP).

[38] Szegedy C., Liu W., Jia Y., Sermanet P., Reed S., Anguelov D., Erhan D., Vanhoucke V., Rabinovich A. Going deeper with convolutions. Proceedings of the IEEE Conference on Computer Vision and Pattern Recognition.

[39] Mettes P., Koelma D.C., Snoek C.G. The imagenet shuffle: Reorganized pre-training for video event detection. Proceedings of the 2016 ACM on International Conference on Multimedia Retrieval.

[40] Kay W., Carreira J., Simonyan K., Zhang B., Hillier C., Vijayanarasimhan S., Viola F., Green T., Back T., Natsev P. (2017). The kinetics human action video dataset. arXiv.

[41] Ye M., Guo Y. Progressive ensemble networks for zero-shot recognition. Proceedings of the IEEE/CVF Conference on Computer Vision and Pattern Recognition.

[42] Soomro K., Zamir A.R., Shah M. (2012). UCF101: A dataset of 101 human actions classes from videos in the wild. arXiv.

[43] Van Der Maaten L. (2014). Accelerating t-SNE using tree-based algorithms. J. Mach. Learn. Res..

